# Changes in quality-adjusted life expectancy in Belgium, 2013 and 2018

**DOI:** 10.1186/s13690-022-01011-0

**Published:** 2022-12-17

**Authors:** Aline Scohy, Rana Charafeddine, Lisa Van Wilder, Herman Van Oyen, Delphine De Smedt, Brecht Devleesschauwer

**Affiliations:** 1 Lifestyle and chronic diseases, Department of Epidemiology and Public Health, Sciensano, Rue J Wytsman 14, 1050 Brussels, Belgium; 2 Department of Public Health and Primary Care, Ghent University, Ghent, Belgium; 3 Department of Translational Physiology, Infectiology and Public Health, Ghent University, Merelbeke, Belgium

**Keywords:** Health-related quality of life, Life expectancy, Health expectancy, Quality-adjusted life expectancy, EQ-5D

## Abstract

**Introduction:**

No information is available in Belgium on life expectancy adjusted for health-related quality of life (HRQoL). Quality-adjusted life expectancy (QALE) captures the multidimensionality of health by accounting for losses in mortality and HRQoL linked to physical, mental, and social impairments. The objective of this study is to estimate for Belgium QALE, the changes in QALE between 2013 and 2018 and the contribution of mortality, HRQoL and its dimensions to this trend.

**Methods:**

The Belgian Health Interview Survey (BHIS), a representative sample of the general population, included the EQ-5D-5L instrument in 2013 and 2018. The tool assesses HRQoL comprising five dimensions (mobility, self-care, usual activities, pain/discomfort, anxiety/depression) using a 5-level severity scoring to define a large variety of health states. The Sullivan method was used to compute at different ages QALE by gender using mortality data from the Belgian statistical office and average EQ-5D scores from the BHIS. QALE was calculated for 2013 and 2018, and changes in QALE over time were decomposed into mortality and ill-health effect.

**Results:**

In 2018, QALE at age 15 years (QALE_15_) was 56.3 years for women and 55.8 years for men, a decrease from 2013 by 0.7 year for women and a stagnation for men. In men, the decrease in mortality counterbalanced the decline in HRQoL. The decline in QALE in women is driven by a decrease in mortality rates that is too small to compensate for the substantial decline in HRQoL before the age of 50 years. In women at older ages, improvements in HRQoL are observed. In women, QALE_15_ is decreasing due to an increase in pain/discomfort, anxiety/depression and problems in usual activities. In men at age 15, the pain/discomfort and anxiety/depression domains contributed to the stagnation. QALE_65_ increased somewhat, due to an improvement in self-care and mobility for both genders, and usual activities and anxiety/depression in men only.

**Conclusion:**

The strength of QALE as member of the family of composite indicators, the health expectancies, is the multidimensional structure of the underlying health component, including both ill-health with different health domains as levels of severity. The ability to decompose differences in the health expectancy not only into a mortality and health component but also into the different health dimensions allows to better inform on general population health trends. Next, compared to other health expectancy indicators, QALE is more sensitive to changes at younger ages.

**Supplementary Information:**

The online version contains supplementary material available at 10.1186/s13690-022-01011-0.

## Introduction

Summary measures of population health (SMPH) combine information on mortality and morbidity. They are essential to better assess the health and changes in health of populations. There is a family of SMPH based on the different definitions of health, and the method on how the health impact of non-fatal outcomes are accounted for. Within the “expectation” measures, the healthy life years (HLY) is, within Europe, the most used disability-free life expectancy indicator (DFLE). HLY uses the global activity limitation indicator (GALI) instrument as underlying measure. GALI is a single-item measure of participation restriction where individuals are asked to rate long-term health-related restrictions in usual activities with three degree-of-difficulty responses. It has been validated in Belgium and in the European context among others, by comparing the results with functional limitations indicators and negative health outcomes [1–4]. Although different health dimensions should be implicitly embedded within GALI, it has been shown that the GALI may not fully grasp ill mental health as e.g. assessed by the GHQ-12 instrument, a self-administered screening questionnaire for common mental disorders [5].

Health-related quality of life (HRQoL) is a multi-dimensional concept that captures the impact of health status on quality of life and includes self-reported physical, mental and social functioning perceptions [6, 7]. Several instruments can be used to elicit HRQoL in a population. The Belgian Health Interview Survey (BHIS) included in the last two waves (2013, 2018) the self-administered EQ-5D-5L instrument developed by the EuroQol group [8]. The EQ-5D-5L is a preference-based standardised measure of self-perceived health status. HRQoL measured by EQ-5D has various strengths. First, the EQ-5D-5L is composed of a self-reported questionnaire consisting of five dimensions (mobility, self-care, usual activity, pain/discomfort, anxiety/depression) with five responses related to severity levels (no problems, slight problems, moderate problems, severe problems and extreme problems/unable to). This multidimensionality allows the assessment of not only physical impairments and activity limitations but also mental health, pain, and social domains. While physical impairments and activity limitations are linked with higher age, ill mental health and musculoskeletal complaints are increasing rapidly already early in life. Several studies have shown the importance of mental health problems in the younger population and their costs to society [9, 10]. The Belgian burden of disease study computed the conditions with the highest disability-adjusted life years (DALY) in 2018. Among people aged 15–44 years, mental and substance use disorders and musculoskeletal disorders were the leading causes of DALYs [11]. Given the growing mental health awareness, measures integrating more than physical impairments are of increasing importance.

Secondly, HRQoL accounts for a large number of health states (vs binary indicators). A health state is defined by the combination of one level for each of the five dimensions. In total, 3125 different health states are possible, based on all the possible response combinations [8].

Thirdly, each health state is weighted by preferences estimated within a general population. The use of population preferences allows summarizing health states based on several questions into a single metric (i.e. the index value) [8]. An EQ-5D-5L value set based on the preferences estimated within the Belgian population was recently developed by the Belgian Health Care Knowledge Centre (KCE) [12].

Finally, the information on HRQoL can be used as the health component in combination with mortality rates to estimate the years lived in different HRQoL states: the quality-adjusted life expectancy (QALE). QALE has been suggested as a relevant measure to summarize the health of a population, monitor changes over time, and compare regions considering different health dimensions, mental and social health as well as physical health.

QALE has already been computed in several countries, including the US [13–15], England [16–18], the Netherlands [19], and in South-Korea [20]. Currently, however, no estimates on life expectancy adjusted for HRQoL are available for Belgium. The objective of this study is not only to assess QALE in Belgium for the years 2013 and 2018 but also to decompose the changes in QALE between 2013 and 2018 at different ages by its mortality and health effect and to identify driving health domains with the health effect.

## Methods

Computation of QALE requires mortality and HRQoL data. We obtained mortality rates by age from Statistics Belgium [21].

### HRQoL data

The BHIS is a cross-sectional household survey that includes a representative sample of the Belgian population through multistage stratified sampling. It included 10,829 respondents in 2013 and 11,611 in 2018 [22–24]. In both surveys, only participants 15 years and older replied to the EQ-5D-5L instrument.

The replies of each participant on the five dimensions of the EQ-5D-5L tool are combined into one score (the HRQoL score or index value) using the Belgian value set of population preferences [12]. Bouckaert et al. described the method to obtain the value set related to the different health states [12]. In short, the EQ-5D-5L Belgian value set was developed in 2021 using data from a survey conducted in a representative sample of the Belgian population. The value set gives an HRQoL score for each combination of responses on the five dimensions and the related severity states. The HRQoL scores range from − 0.532 (worst health state) to 1 (perfect health state) [25]. For example, the HRQoL score of someone reporting moderate problems with anxiety/depression and no other problem is 0.849, and the HRQoL score is 0.678 for a person reporting moderate problems in usual activity, pain/discomfort, and anxiety/depression.

The HRQoL scores were applied to BHIS respondents. Population norms, i.e. average HRQoL score by age, gender, and region, have been computed by Van Wilder et al. [7]. The average HRQoL score in 2018 for men aged 15 years was 0.92 and for women was 0.89. The population norms represent the average score of the self-perceived health status based on the 5 dimensions of the EQ-5D-5L and their related severity levels for a specific age, gender and region. To assess the contribution of each of the domains on the HRQoL, each dimension was dichotomised into no problem versus any problems.

### QALE

QALE was computed using the Sullivan method to compute health expectancies [26]. This method deflates the number of person-years according to health states, and then adds those person-years and calculates the life expectancy in a health state as in the standard life table. We used non-abridged life tables with an open last age group at age 100. The morbidity part was defined by the population norms, i.e. the average age, gender and region HRQoL score based on the 5 dimensions of the EQ-5D-5L. The population norms were applied to the respective life table person-years [19]. QALE at different ages was calculated by gender, and region (Flemish Region, Brussels-Capital Region, Walloon Region), based on the stratum-specific mortality rates and EQ-5D-5L population norms. QALE starts at 15 years because HRQoL for children was not assessed in the BHIS. Standard errors were calculated and statistical significance between two health expectancies was conservatively tested by a Z-score based on the method developed by the EURO-Reves team [26]. Next, we computed life expectancies for the probability of reporting no problems for each dimension separately. The prevalence of reporting no problem for each dimension was applied to the respective life table person-years. We focused here on results at 15 years and 65 years of age.

### Decomposition of QALE

The method developed by Nusselder and Looman was used to decompose the change in QALE between 2013 and 2018 into its mortality and HRQoL part [27]. The method estimates the part of the change attributed to the mortality effect (${}_i{}{MOR}_x$), i.e., the change in the number of person-years adjusted by HRQoL due to the change in person-years lived (${}_i{}{L}_x$) (Eq. 1), and the disutility effect (${}_i{}{DIS}_x$), i.e. the change in the number of person-years adjusted by HRQoL due to a change in the health component, the population norms (${}_i{}{\pi}_x$) (Eq. 2). The sum of both effects corresponds to the change between two QALE values [27].

1 $${}_i{}{MOR}_x=\left(\frac{{}_i{}\pi_{x(t)}+{}_i{}\pi_{x\left(t+n\right)}}2\right)\;\cdot\;\Delta_iL_x$$

2 $${}_i{}{DIS}_x=\left(\frac{{}_i{}L_{x(t)}+{}_i{}L_{x\left(t+n\right)}}2\right)\;\cdot\;\Delta_i\pi_x$$

For each age group, the differences between QALE calculated in 2013 and in 2018 for men and women were decomposed.

All calculations were performed in R 4.1.0 [28]. Belgian population norms are available through the EQ5D.be R package [7].

## Results

### Quality-adjusted life expectancy

In 2018, QALE at 15 years (QALE_15_) in Belgium was 55.8 for men and 56.3 for women. Between 2013 and 2018, QALE_15_ stagnated for men and decreased for women (− 0.7 year, *p* <  0.001). QALE_65_ slightly increased between 2013 and 2018 for both genders (p <  0.001), and women experience one additional year of QALE_65_ than men (Table 1). Supplementary file 1 and 2 provide detailed information on QALE by age, gender, and region for both periods. Regional information is important in the Belgian context but these results will not be commented on in the text.

**Table 1:** Life expectancy (LE), quality-adjusted life expectancy (QALE), and LE without problems in each of the five dimensions at age 15 and 65 years, by gender, 2013 and 2018, Belgium

Age	Gender	Year	LE	QALE	LE without problems				
					*No problems on Mobility*	*No problems on Self-care*	*No problems on Usual activities*	*No problems on Pain and discomfort*	*No problems on Anxiety and depression*
15	Men	2013	63.5	55.8	52.9	59.3	53.4	34.4	49.5
		2018	64.6	55.8	53.2	60.8	54.3	31.6	48.8
		Change	+ 1.1	0.0	+ 0.3	+ 1.5	+ 0.9	−2.8	−0.7
		*P*-value		0.304	0.313	0.186	0.049	< 0.001	0.051
	Women	2013	68.6	57.0	53.1	61.8	53.6	31.8	47.8
		2018	69.1	56.3	52.9	63.6	52.2	26.6	43.2
		Change	+ 0.5	−0.7	−0.2	+ 1.8	−1.4	−5.2	−4.6
		*P*-value		< 0.001	0.349	0.128	0.006	< 0.001	< 0.001
65	Men	2013	17.6	14.6	11.3	14.6	12.5	7.4	13.4
		2018	18.4	14.7	11.6	15.7	13.2	6.3	14.3
		Change	+ 0.8	+ 0.1	+ 0.3	+ 1.1	+ 0.7	−1.1	+ 0.9
		*P*-value		< 0.001	0.013	< 0.001	< 0.001	< 0.001	< 0.001
	Women	2013	21.1	15.6	11.3	16.1	13.1	6.5	14.8
		2018	21.6	15.7	11.7	17.6	12.8	4.9	13.2
		Change	+ 0.5	+ 0.1	+ 0.4	+ 1.5	−0.3	−1.6	−1.6
		*P*-value		< 0.001	0.107	< 0.001	0.055	< 0.001	< 0.001

**Fig. 1:**
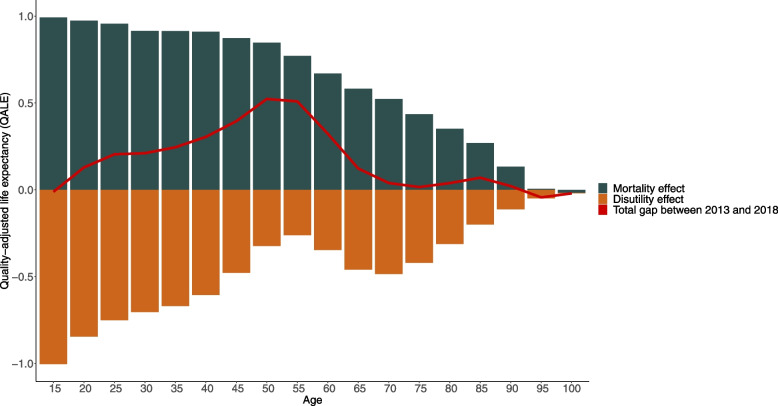
Decomposition of differences in quality-adjusted life expectancy at different ages in men between 2013 and 2018, Belgium

**Fig. 2:**
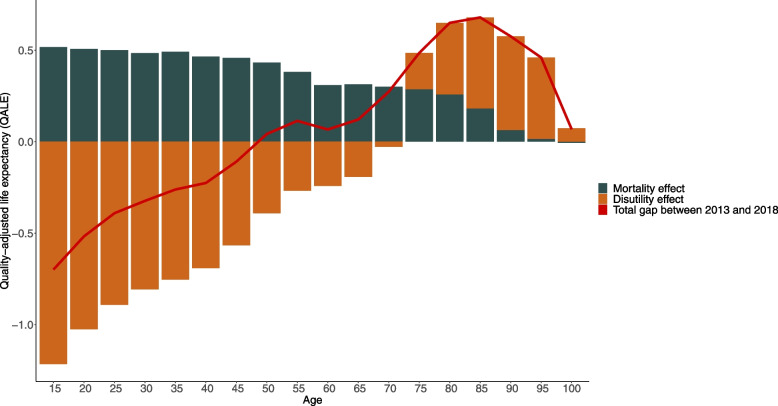
Decomposition of differences in quality-adjusted life expectancy in women at different ages between 2013 and 2018, Belgium

The differences in QALE between 2013 and 2018 were broken down for each age group by gender to determine which part of the change is attributable to the change in the age and gender-specific mortality rates (mortality effect) and to the change in age and gender-specific HRQoL index score (disutility effect). The decomposition showed that the decrease in QALE was driven by a worsening HRQoL. In men, there was no difference in QALE_15_ (red line) as the 1-year increase due to the decrease in mortality rate (black bar) was nullified by a 1-year decrease due to the decline in HRQoL (orange bar) between 2013 and 2018 (Fig. 1). During adulthood, QALE is higher in 2018 than in 2013 as the positive mortality effect was more substantial than the effect of the declining HRQoL. At age 65 years, QALE was 0.1 year higher in 2018 than in 2013. This results from an increase of 0.6 year due to the mortality effect and a decrease of 0.5 due to the loss in HRQoL. In women, at 15 years the difference in QALE between 2013 and 2018 was − 0.7 year. This is the result of an increase of 0.5 year due to the decrease in mortality rates and of a decrease of 1.2 years due to the loss in HRQoL. The overall decline in QALE_15_ in women is driven by the too small decrease in mortality rates that was insufficient to compensate for the important decline in HRQoL up to the age of 50 years (Fig. 2). However, important improvements in HRQoL are observed at higher ages in women. E.g. at 75 years old, QALE is increasing by 0.5 year due to the mortality effect (+ 0.3 year) and to the increase in HRQoL (+ 0.2 year).

### The EQ-5D-5L dimensions

Barely any changes were observed between 2013 and 2018 in the life expectancy without problems in mobility, and men and women experienced a comparable number of years at 15 and at 65 years. In 2018, men and women at age 15 years experienced 60.8 and 63.6 years respectively without problems in self-care. This is a progress of 1.5 (*p* = 0.186) and 1.8 years (*p* = 0.128) compared to 2013. At 15 years, the number of years without problems in usual activities increased among men (+ 0.9 year, *p* = 0.049), and decreased among women (− 1.4 years, *p* = 0.006). At 15 years, the number of years without problems in pain and discomfort decreased between 2013 and 2018. Men lived 31.6 years and women 26.6 years without pain/discomfort, a decrease of 2.8 years and 5.2 years (*p* <  0.001), respectively, compared to 2013. At 15 years, men experienced more years (48.8) without problems of anxiety and depression than women (43.2). For men at 15 years, the number of years without anxiety/depression decreased between 2013 and 2018 (− 0.7 year, *p* = 0.051), but at 65 years this number increased over time (+ 0.9, p <  0.001); whereas, in women we saw an important decrease in the number of years without anxiety and depression (− 4.6 years at 15 and − 1.6 years at 65, p <  0.001) (Table 1).

**Fig. 3:**
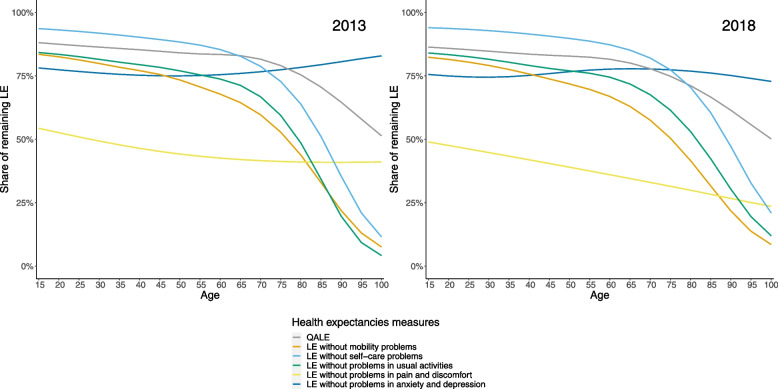
Ratio quality-adjusted life expectancy and life expectancy without problems on the five dimensions compared to life expectancy between 15 and 100 years old, men, Belgium, 2013 and 2018

**Fig. 4:**
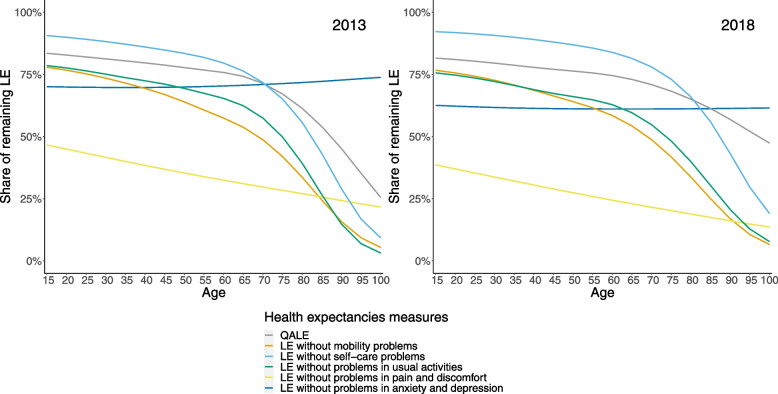
Ratio quality-adjusted life expectancy and life expectancy without problems on the five dimensions compared to life expectancy between 15 and 100 years old, women, Belgium, 2013 and 2018

In 2018, at 15 years, men lived only half of their remaining life expectancy without problems in pain and discomfort (Fig. 3), while women lived only 40% of their remaining years without problems in pain and discomfort (Fig. 4). In contrast to the other dimensions, the share of the remaining life expectancy to be spent without problems in anxiety and depression does not decrease throughout the age groups. Reduced mobility, usual activities, and self-care are dimensions that impact remaining life expectancy mainly at older ages and impact women more than men. In general, men lived more of their remaining life expectancy in good QALE than women at all ages.

While men lived a similar share of their remaining life expectancy in good QALE in 2013 and 2018, the remaining life expectancy in good QALE was higher in 2018 than in 2013 for women at older age.

## Discussion

We computed QALE estimates for Belgium for the years 2013 and 2018 and decomposed the changes in QALE into a mortality and health effect. In addition, we investigated which of the different health dimensions were drivers of the changes in QALE. The evolution in QALE_15_ and QALE_65_ between 2013 and 2018 provides evidence that the gain in life expectancy over this period was not translated into a gain in health expectancy.

In 2018, QALE_15_ was 56.3 years for women and 55.8 years for men, a decrease from 2013 by 0.7 year for women and a stagnation for men. In men, the decrease in mortality counterbalanced the decline in HRQoL. The decline in QALE_15_ in women is driven by a decrease in mortality rates that is too small to compensate for the substantial decline in HRQoL before the age of 50 years. In women at older ages, improvements in HRQoL are observed. In women, QALE_15_ is decreasing due to an increase in pain/discomfort, anxiety/depression and problems in usual activities. In men at age 15, the pain/discomfort and anxiety/depression domains contributed to the stagnation. QALE_65_ increased somewhat, due to an improvement in self-care and mobility for both genders, and usual activities and anxiety/depression in men only.

Our results show the possibility to get insights into the effect of different domains. QALE includes not only physical impairments and activity limitations but also complaints and mental health in the health component. We observed diverging trends by dimensions, gender and age. Whereas dimensions linked to physical impairments and activity limitations (in men) showed progress, the dimensions pain/discomfort and anxiety/depression showed worsening. These dimensions are also the dimensions where gender inequalities are particularly important in disfavour of women [7].

It provides insights into the effect of different domains but also on their severity levels. The health component of QALE evaluates a large variety of health states valuated by population preferences, contrary to measures such as the DFLE where the health component is binary. As observed, the share of the remaining life expectancy to be spent in good QALE is higher than the share of remaining life years without problems in mobility, self-care, usual activities, and pain and discomfort. The DLFE for the specific dimensions is lower, especially at higher age, because people increasingly report having some level of problems. However, these problems can be valuated as not resulting in substantial HRQoL loss by the population, so QALE will not decrease as much as the dimension-specific DFLE.

Another strength of QALE is that it is more sensitive to ill-health states at younger ages. The observed decrease in QALE_15_ in women is the result of worsening in all health domains at young ages except self-care and especially in the pain/discomfort and anxiety/depression domains. Ill-health at young age is worrisome as it increases the risk for ill-health throughout life. For example, younger individuals with mental ill-health are at increased risk for chronic mental disorders (e.g. substance use disorders and anxiety disorders) and disabling physical conditions in later life [9, 29]. The results at 65 years old showed that progress has been made, particularly in the self-care dimension, but there is a negative trend of pain/discomfort in both genders and of anxiety/depression among women.

Some limitations have to be considered. International comparisons in QALE are seriously hampered due to the use of country-specific valuation sets. It has been shown that applying valuation sets from different countries leads to important differences in QALE estimates (maximum difference of 7.2 years) and changes in the ranking of average performer countries [30]. Only a few countries computed QALE for the general population, including the US [13–15], England [16–18], the Netherlands [19], and South Korea [20], and some used different instruments to valuate HRQoL. Interestingly, the dimensions pain/discomfort and anxiety/depression were the main drivers of inequalities in HRQoL between deprivation quintiles in the North-West of England [18].

Furthermore, we observed the evolution in QALE dimensions by looking at life expectancy without problems for the five dimensions. This analysis by health domain ignores the severity levels. While it brings valuable information on the evolution of health domains, differences in the prevalence of each dimension may not represent the evolution of severity distribution.

This study used mortality data for the entire Belgian population and population norms based on HRQoL data for a large and representative sample of the population. However, certain limitations in the estimations of population norms also applied to our study [7]. A selection bias would lead the HRQoL results to be biased towards a more healthy population as severely impaired people would not have been able to answer the self-reported questionnaire. Moreover, the health status is not fixed in one person and could fluctuate over time, particularly for people with chronic conditions. While in some chronic conditions health states are relatively constant, in other chronic conditions, e.g. migraine, multiple sclerosis, and epilepsy, patients may experience episodes with stronger symptoms [31, 32]. The EQ-5D instrument asks about the health status ‘today’ but it has been shown that people effectively used different recall periods and times perspectives to answer the tool, particularly if they have fluctuating health states [32, 33].

One additional limitation of our analysis is that QALE could only be computed from 15 years old. Estimating and interpreting HRQoL in children is complex. A youth version of the EQ-5D is available for children from 8 years and above (EQ-5D-Y). However, existing valuation sets cannot be assumed to be valid for EQ-5D-Y [34].

Future studies may explore inequalities in QALE by computing QALE by socioeconomic groups [16, 18, 19] or at provincial level [17]. Furthermore, continuing to include the EQ-5D in future BHIS will allow us to monitor the changes in QALE over time.

## Conclusion

The negative evolution of QALE is due to the increase in years in reduced HRQoL. The improvements in the reduction of mortality counterbalanced the decrease of HRQoL in men but did not compensate for the decrease in HRQoL in women before 50 years old. In women, QALE_15_ is decreasing due to increasing problems in pain/discomfort and anxiety/depression. Ill-health is already present at young age and this did not improve. QALE_65_ remained unchanged or increase somewhat due to a reduction of problems in self-care and mobility for both genders, and in usual activities and anxiety/depression for men.

## Supplementary Information

**Additional file 1: Supplementary file 1.** QALE and life expectancy with no problems in each of the 5 dimensions for the Belgian population aged 15 years and older in 2013, by age, gender, and region.

**Additional file 2: Supplementary file 2.** QALE and life expectancy with no problems in each of the 5 dimensions for the Belgian population aged 15 years and older in 2018, by age, gender, and region.

## Data Availability

The data on mortality used in the current study is available on the website of the Belgian statistical office, Statbel. https://statbel.fgov.be/fr/themes/population/mortalite-et-esperance-de-vie/tables-de-mortalite-et-esperance-de-vie#figures The data on population norms used in the current study is available through a R package, EQ5D.be. https://github.com/brechtdv/EQ5D.be

## Abbreviations

BHIS: Belgian Health Interview Survey
DFLE: Disability-Free Live Expectancy
GALI: Global Activity Limitation Indicator
HLY: Healthy Life Years
HRQoL: Health-Related Quality of Life
LE: Life Expectancy
QALE: Quality-Adjusted Life Expectancy
SMPH: Summary Measure of Population Health
UK: United Kingdom
